# Personal

**Published:** 1899-02

**Authors:** 


### PERSONAL.

DR. OSWALD C. STACKHOUSE, of Buffalo, was recently appointed assistant physician at the Hudson River State Hospital at Poughkeepsie.

---

DR. JOHN H. GRANT, of Buffalo, who was appointed an acting assistant surgeon U. S. Army and assigned to duty at the U. S. General Hospital, at Fort McPherson, (Atlanta, Ga.,) has recently returned. He has retired from the military service and resumed his practice as well as his duties as milk inspector in the Department of Health.

---

DR. FREDERICK HOYER MILLENER has announced that he has removed his office to No. 5 North Pearl street, where he will continue to treat diseases of the nose, throat and ear as a specialty. Hours: 9 a. m. to 1 p. m.; 7 to 8 p. m. Other hours by appointment.

Dr. William H. Mountain, who has been house surgeon at the Emergency Hospital for the last six months, has been appointed resident physician at the Buffalo Hospital of the Sisters of Charity. Dr. William T. Griffin of the Sisters' Hospital has taken Dr. Mountain's place as house surgeon at the Emergency.

---

Dr. Edward J. Ill, of Newark, N. J., president-elect of the American Association of Obstetricians and Gynecologists for the meeting of the third week in September, to be held in Indianapolis, was the guest of Dr. L. H. Dunning, December 13th and 14th, at Indianapolis.

Dr. Dunning gave him a dinner at the Denison House from 6 to 8 p. m., where twenty or more of the physicians of the city, representing each of the varied medical interests and institutions, had the pleasure of meeting and conversing with Dr. Ill.

After the supper the visitor and guests attended the meeting of the Marion County Society, where Dr. Dunning read an essay on Some minor disorders of the female genitalia, including kraurosis vulvæ, urethral caruncle and senile endometritis, which was discussed by Drs. Ill, Pantzer, Eastman, Pfaff and others.

The following day Dr. Ill operated at the Indianapolis City Hospital clinic on a case of parovarian cyst. Dr. Dunning also operated upon a case of double papillomatous cyst, with rupture, secondary involvement and ascites. It is gratifying to state that two weeks have elapsed since the operations, and both patients have gone on without accident, and are virtually recovered. This is the more noteworthy as it is a well-known fact that operators off their native heath have so often been unfortunate in the outcome of their illustrative operations that there is a very considerable and growing timidity on their part in operating outside their own hospitals and sanitariums.

Dr. Ill has been kind enough to forward this journal a report of his case, which is published in this issue in connection with Dr. Dunning's case.

It is needless to say that Dr. Ill is a genial and attractive character, quiet and unobtrusive, as good a listener as a converser, and that the profession in Indianapolis was pleased at the opportunity of making his acquaintance.—*Indiana Medical Journal*, January.

---

Major George R. Fowler, of Brooklyn, Chief Surgeon U. S. Volunteers, is honorably discharged, to take effect February 1, 1899.

Dr. Fowler has been with the 7th Army Corps, General Fitzhugh Lee commanding, in Cuba. He is one of the state medical examiners and he will be warmly welcomed home by his colleagues on the board.

